# Physical and Chemical Characteristics of *Aedes aegypti* Larval Habitats in Nouakchott, Mauritania

**DOI:** 10.3390/tropicalmed10060147

**Published:** 2025-05-23

**Authors:** Mohamed Haidy Massa, Mohamed Aly Ould Lemrabott, Osman Abdillahi Guedi, Sébastien Briolant, Ali Ould Mohamed Salem Boukhary

**Affiliations:** 1 Unité de Recherche Génomes et Milieux (GEMI), Université de Nouakchott, Nouveau Campus Universitaire, Nouakchott BP 5026, Mauritania; medhaidy@gmail.com (M.H.M.); mohamedalylemrabott@yahoo.fr (M.A.O.L.); alimedsalem@gmail.com (A.O.M.S.B.); 2 Département des Sciences Humaines et Sociales, Faculté de Lettres, Langues et Sciences Humaines et Sociales, Université de Djibouti, Campus de Balbala, Croisement RN2-RN5, Djibouti 1904, Djibouti; osman.guedi@gmail.com; 3 Département de Géographie, Université de La Réunion, 97744 Saint-Denis, France; 4 Unité Parasitologie et Entomologie, Département Risques Vectoriels, Institut de Recherche Biomédicale des Armées (IRBA), 13005 Marseille, France; 5 Service de Santé des Armées, Assistance Publique-Hôpitaux de Marseille, Aix Marseille University, Risques Infectieux Tropicaux et Microorganismes EmergentS, 13005 Marseille, France; 6 Institut Hospitalo-Universitaire Méditerranée Infection, 13005 Marseille, France

**Keywords:** *Aedes aegypti*, arbovirus, breeding sites, larvae, Mauritania, Nouakchott

## Abstract

*Aedes aegypti*, the main urban vector of dengue fever, represents a growing public health problem in Nouakchott, the capital of Mauritania. Identifying the factors influencing the distribution and productivity of its breeding sites is essential for the development of effective control strategies. From May 2023 to April 2024, physico-chemical characteristics were recorded and mosquito larvae were collected, using a standard dipping method, from 60 water collections each month during the dry season and twice a month during the rainy season, totaling 294 observations. The larval positivity of water collections and larval abundance of breeding sites over the time were modeled using a random-effect logistic regression model and a negative binomial regression model, respectively. The depth, distance from habitat, type of water collection and exposure to sunlight were statistically significant and independently associated with water collection positivity for *Ae. aegypti* larvae (aOR = 5.18, 95%CI [1.66–16.18], *p*-value = 0.005; aOR = 0.00, 95%CI [0.00–0.02], *p*-value < 0.001; aOR = 252.88, 95%CI [4.05–15,786.84], *p*-value = 0.009 and aOR = 0.04, 95%CI [0.01–0.26], *p*-value < 0.001, respectively). *Aedes aegypti* larval habitats were mainly artificial (90%), temporary (*n* = 217 observations), close to dwellings (*n* = 114) and shaded (*n* = 96). Plastic water tanks (*n* = 17, 48.6%), wells (*n* = 6, 17.1%) and barrels (*n* = 4, 11.4%) were the most common breeding sites. Larval abundance was negatively associated with containers of increasing pH and surface area (aOR = 0.50, 95%CI [0.33–0.75] *p*-value = 0.001 and aOR = 0.48, 95%CI [0.27–0.87], *p*-value = 0.016, respectively). As *Ae. aegypti* mosquitoes are multi-resistant to adult insecticides and dengue has become endemo-epidemic since 2014, vector control should give the priority to the physical removal or treatment of shaded, peridomestic containers—particularly plastic water tanks and barrels—and consider the use of biological larvicides to target breeding sites with low pH and small surface areas.

## 1. Introduction

*Aedes aegypti* is the main urban mosquito vector of dengue virus and other highly pathogenic human arboviruses, such as yellow fever virus, chikungunya virus and Zika virus [1]. According to the WHO global dengue surveillance, 8,265,077 confirmed dengue cases including 56,495 severe cases, and 11,819 deaths were reported between January 2024 and March 2025 [2]. The transmission of arboviruses depends on multiple factors, such as mosquito blood-feeding preferences, the productivity of breeding sites and the density of adult mosquitoes, which also depend on meteorological variables in a context of climate change [3]. The spread of *Aedes* mosquitoes has transcended geographical barriers, reaching many parts of the globe. Dengue is estimated to be endemic in approximately 90 countries [4] while also being present in others. As a result, people are infected with different types of arboviruses, thus increasing the prevalence of arboviral diseases worldwide [5].

*Aedes aegypti* is well adapted to the urban environment and lays its eggs almost exclusively in small artificial containers with relatively clean water [6]. These included tires, flower pots, drains, and water tanks [6,7]. However, albeit rarely, some studies have reported the presence of *Ae. aegypti* larvae thriving in natural breeding sites, such as tree holes, within urban environments [8]. Water quality is essential for the selection of a particular larval habitat by gravid females to ensure egg hatching and the development of their offspring from larvae to adults [9,10,11]. Females select breeding sites according to the abiotic and biotic elements (e.g., organic matter) present in the water [9]. Therefore, the physicochemical characteristics of larval habitats, including water salinity, pH, conductivity and dissolved solids, are considered key factors in female mosquito oviposition and larval development [12]. Salinity and pH are considered the most important for mosquito presence [13]. In addition, larger breeding sites with a greater volume of water are more productive [9]. Furthermore, exposure to sunlight is a key factor influencing the productivity of *Ae*. *aegypti* in different water collections [14].

The most effective vector control strategy is to eliminate breeding sites or kill larvae [15]. It requires in-depth knowledge of the larval ecology of the species concerned, whereas chemical control, which consists of reducing the adult mosquito population, can lead to resistance, as has already been reported with pyrethroid resistance in *Ae. aegypti* in Nouakchott [16]. In the case of *Ae. aegypti*, whose activity is diurnal and crepuscular [17], impregnated mosquito nets are useless for preventing DENV transmission except for symptomatic patients with viremia. *Aedes aegypti* is anthropophilic [18] and often breeds in domestic and peridomestic artificial containers [19], but the typology of breeding sites and their productivity can vary both between countries and between localities within the same country [20,21].

The first documented dengue outbreak in Nouakchott, the capital city of Mauritania, occurred during October–November 2014 [22]. Prior to this outbreak, there were no documented cases of dengue fever in Mauritania. Entomological surveys conducted in mid-2014 revealed the presence of *Aedes aegypti* mosquitoes in Nouakchott, marking the first detection of this primary dengue vector in the country [7]. Following the 2014 epidemic, dengue outbreaks have been reported annually in Nouakchott and other urban settings [23]. These epidemics have exerted significant pressure on the populations, healthcare systems and economies of the majority of tropical countries worldwide [24]. In the absence of a commercial dengue vaccine and effective therapies, vector control remains the most effective strategy for limiting transmission. This includes continuous vector surveillance, the integrated management of *Aedes* mosquitoes using safe and cost-effective biological and chemical controls, environmental management, legislation and measures at individual and community levels [25].

In Mauritania, among mosquitoes of medical importance, only the ecology of *Anopheles* larvae has been studied [26]. The main objective of the present study was to describe the physicochemical characteristics of water collections associated with positivity for *Ae. aegypti* larvae in Nouakchott in order to assist health authorities in the prevention of arboviruses transmission.

## 2. Materials and Methods

### 2.1. Study Site and Period

This study was carried out in Nouakchott, the capital of the Islamic Republic of Mauritania and one of the largest cities in the Saharan region. It is located along the Atlantic coast and characterized by a low altitude ranging from 1 m to 10 m below sea level. A natural salt belt 1 to 2 km wide separates the city proper from the Atlantic coast. The climate is generally arid and hot with seasonal variations in rainfall and temperature. The long dry season that characterizes the climate in Nouakchott lasts between September and June, while the short wet season extends from July to September.

From 2000 to 2015, the Nouakchott weather station recorded average annual temperatures of 25.6 °C [27]. The hottest months are June to October, with average temperatures above 27 °C, and January is the coolest with an average of 21.4 °C. The average maximum and minimum temperatures recorded are 33 °C and 20.6 °C, respectively. The average annual temperature over the study period ranged from a low of 21.8 °C to a high of 31.9 °C. Annual relative humidity ranged from 28 to 69%. Precipitation is generally concentrated in the summer months (July, August and September) with an average of 120 mm per year. Winds are multidirectional with a predominance of northeast winds from October to May and northwest winds from June to September for each season of the year [28]. Nouakchott is densely populated with around 1,500,000 inhabitants, comprising nearly one in three Mauritanians living in Nouakchott [29].

This is a cross-sectional entomological survey carried out over a one-year period, from May 2023 to April 2024, in domestic and peridomestic areas to detect mosquito larval development sites. A total of 60 water collections and water-holding containers were visited once a month during the dry season and twice a month during the rainy season. During the study period, rainfall reached 109.5 mm (30 mm in June, 15.5 mm in July, 54 mm in August and 10 mm in September).

### 2.2. Larval Sampling and Morphological Identification of Adults

As part of the entomological monitoring program of *Aedes aegypti* breeding sites, a rigorous, stratified sampling plan was developed to ensure representative coverage of the different ecological conditions likely to influence larval population dynamics. Sampling was stratified along two axes. Firstly, the types of containers observed in the field were taken into account, as they are likely to vary in their propensity to harbor larvae. These included plastic and metal containers as well as well water tanks. Secondly, Moughataa were considered, taking into account reported dengue cases, mosquito density and contextual differences in terms of urbanization and access to water.

Different types of water collection were inspected, and larvae were collected accordingly. Mosquito larvae were collected using a standard dipping method with a mosquito scoop (Bioquip, Gardena, CA, USA) [30]. For containers with small size, larvae and pupae were collected by either pipetting or emptying the container. All mosquito larvae were counted to determine the density. The collected mosquito larvae were transported alive to the insectarium in labeled 750 mL capacity mineral water bottles for rearing under ambient temperature 28 °C and relative humidity 80% with a 12:12 h (light:dark) photoperiod. All emerged adults were aspirated after rearing, placed at −20 °C for a few minutes to kill them and preserved in Eppendorf tubes containing cotton and silica gel. Mosquito adults were then morphologically identified, using a stereo zoom binocular microscope, at the species level based on morphological identification key [31,32].

### 2.3. Characterization of Water Collections

For each water collection, we recorded the physical and chemical characteristics using the form already described by Nebbak et al. [33]: pH, temperature (°C), conductivity (μS/cm), salinity (g/L) and turbidity (Formazin Nephelometric Unit) were determined in the field using a portable HANNA HI (98195) (imLab, Wasquehal, France) according to the manufacturer’s model. Other physical characteristics were recorded: geographical location (GPS), type of water collection, water collection depth (≤0.5 or >0.5 m), water collection size (≤5 or >5 m^2^), distance from habitats (≤10 or >10 m), water transparency (transparent or opaque), vegetation (presence or absence), the presence of larvae, larval density according to Papierok et al. [34], sun exposure (shaded, semi-shaded, sunny), water type (permanent or temporary), and origin of water collection (natural or artificial).

### 2.4. Statistical Analysis

Data were analyzed using R software v4.4.2 [35]. All categorical variables were constructed to have balanced effects between groups. The larval positivity of water collections was analyzed as a dependent variable according to individual water collections characteristics, using a random-effect logistic regression model (with the water collection as a random effect because of the spatialization of the data). First, a descriptive analysis of the independent variables was carried out, and a univariate analysis was performed by entering each independent variable into a logistic regression model. The larval density of breeding sites was analyzed as a dependent variable according to the individual characteristic of the breeding sites, using a negative binomial regression with a random effect. In both logistic and negative binomial regression models, variables were retained for multivariate analysis when their effect had a *p*-value < 0.25. A backward stepwise selection procedure with minimization of Akaike’s information criterion was applied to retain significant independent variables (*p* < 0.05) and their interactions in the final model.

## 3. Results

### 3.1. Description of Aedes aegypti Breeding Sites

**Figure 1:**
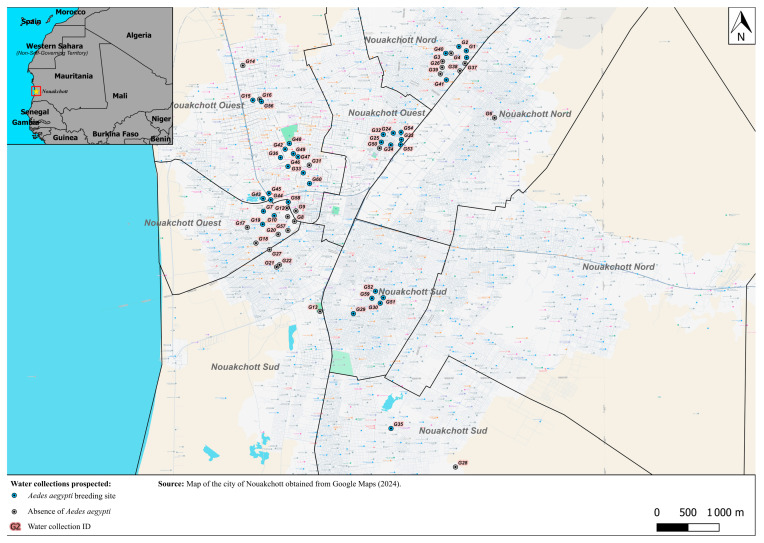
Map showing prospected water collections in Nouakchott. The blue circles indicate the *Aedes aegypti* breeding sites. The red square in the inset indicates the location of Nouakchott.

During the study period from May 2023 to April 2024, 60 water bodies located in the most densely populated residential areas across different districts of Nouakchott were sampled for larvae and analyzed for their physico-chemical characteristics. Each collection was monitored an average of five times with some observed up to six times and others only twice. During the dry season, larval sampling was conducted monthly, while during the rainy season, visits were carried out every two weeks. The geographical location of these water collections is given in Figure 1. Of the 60 water collections prospected, 54 (90%) were artificials and 6 (10%) were naturals. The majority of water pools were temporary (47/60, 78.3%), while the others (13/60, 21.6%) were permanents. Of the 60 water collections visited, 35 (58.3%) were positive for *Ae. aegypti* immature stages (Figure 1, Supplementary Table S1). These included plastic water tanks (*n* = 17, 48.6%), wells (*n* = 6, 17.1%), barrels (*n* = 4, 11.4%), leaking pipes (*n* = 3, 8.6%), puddles of agricultural wastewater (*n* = 2, 5.7%), stagnant rainwater and groundwater (*n* = 1, 2.9%), a standpipe drain (*n* = 1, 2.9%), and an ablution site (*n* = 1, 2.9%). Water pools in Tevragh Zeina district were the most suitable for *Aedes aegypti* larvae with 14 positive water containers, which were followed by those of Ksar district with 8 positive water pools. Photographs of the main *Ae. aegypti* breeding sites are presented as a Supplementary File (Supplementary Figure S1).

### 3.2. Factors Associated with the Positivity of Water Collection for Aedes aegypti Larvae

In order to determine the key factors associated with larval positivity in the water collections surveyed, on the one hand, and larval density in the breeding sites, on the other, entomological data were subjected to regression analyses. Univariate logistic and negative binomial regression analyses are presented in Supplementary Tables S2 and S3.

Univariate logistic regression with random effects analysis of water collection positivity for *Ae. aegypti* larvae showed that temporary artificial shaded containers, located close to households, of sufficient depth (>0.5 m), and containing clear water with low salinity (<0.18 g/L) were significantly associated with habitat infestation by mosquito larvae (Supplementary Table S2).

To provide a clear overview of breeding site characteristics, a comprehensive summary table (Table 1) shows details of sampling sites, water collection types and physico-chemical parameters measured, including pH, temperature, turbidity, salinity, and conductivity.

Supplementary Table S4 shows the distribution of the 294 observations included in the study. It details the number of observations recorded per water collection, allowing a better understanding of the sampling design and the relative representation of each category in the data set.

Multivariate logistic regression with random effect analysis showed that depth, distance from habitat, type of water collection and exposure to sunlight were statistically significantly and independently associated with water collection positivity for *Ae. aegypti* larvae (Table 2). Protective factors were distance to habitat >10 m (aOR = 0.00, 95%CI [0.00–0.02], *p*-value < 0.001) and sun exposure (aOR = 0.04, 95%CI [0.01–0.26], *p*-value < 0.001). Risk factors were depth >0.5 m (aOR = 5.18, 95%CI [1.66–16.18], *p*-value = 0.005) and artificial water collection (aOR = 252.88, 95%CI [4.05–15,786.84], *p*-value = 0.009).

### 3.3. Factors Associated with the Density of Ae aegypti Larvae in Breeding Sites

Multivariate binomial regression with random effect analysis showed that pH and breeding site size were statistically significantly and independently associated with *Ae. aegypti* larval density (Table 3). Both factors were protective: pH ≥ 8.3 (aOR = 0.50, 95%CI [0.33–0.75] *p*-value = 0.001) and size > 5 m^2^ (aOR = 0.48, 95%CI [0.27–0.87], *p*-value = 0.016).

## 4. Discussion

Previous studies have shown that *Ae. aegypti* mosquitoes from Nouakchott are both molecularly [16] and phenotypically resistant to pyrethroids, malathion and bendiocarp [36]. In contrast, *Ae. aegypti* larvae are highly susceptible to the larvicides *Bacillus thuringiensis israelensis* and temephos [36]. Consequently, controlling *Ae. aegypti* larvae remains one of the most effective measures for preventing arbovirus transmission in Nouakchott. To achieve this, it is essential to understand the main characteristics of *Ae aegypti* breeding sites. In our study, the most common type of *Ae. aegypti* larval habitat was artificial. This is consistent with observations from several studies in Burkina Faso [37], Côte d’Ivoire [38], Benin [39], Sri Lanka [40], Saudi Arabia [41], and Brazil [13]. Indeed, plastic tanks, wells and barrels were the most frequent artificial breeding sites as already reported in other studies carried out in urban areas in Congo and Sri Lanka, respectively [40,42]; barrels were also the most common larval habitat observed in a study carried out in Ethiopia [43]. This result differs from those described in Mozambique and Burkina Faso, where used tires were the most frequent breeding sites of *Ae. aegypti* [37,44]. *Aedes aegypti* larvae were observed only once out of 294 observations in natural waters, i.e., rainwater and groundwater. Similar results have been reported in Benin where only 2.5% of *Ae. aegypti* breeding sites surveyed were natural [45].

In addition, a significant proportion of water containers (96 vs. 20) were in the shade. Previous studies have shown that *Ae. aegypti* females prefer shaded containers [46,47]. This association between positive breeding sites for *Aedes* larvae and shade may be due to the fact that shade helps protect larvae from high temperatures by reducing water temperature [15], and it also prevents breeding sites from drying out, thus increasing larval survival [21].

Depth and distance from habitat were statistically significantly and independently associated with water collection positivity for *Ae. aegypti* larvae. This is consistent with several studies carried out in Bangladesh [48] and Burkina Faso [37], where large containers are, after tires, the most common breeding sites for *Ae. aegypti*. The distance of breeding sites from the habitat is also associated with the positivity of a collection water for *Ae. aegypti* larvae. This could be explained by its low average flight distance according to mark-recapture experiments, which is less than 100 m [49], and its strong anthropophily [18]. Indeed, *Ae. aegypti* specifically bites humans and hatches in artificial breeding sites.

In the present study, the *Ae. aegypti* larval density in the city of Nouakchott was negatively associated with pH and breeding site size, as were the results of another study regarding both *Ae. aegypti* and *Ae. albopictus* larvae in the city of Ouagadougou in Burkina Faso [37]. Although another study in India found a positive correlation between *Ae. albopictus* larval abundance and pH [47]. Another study, carried out in Bangladesh, also showed that the density of *Ae. aegypti* larvae was positively associated with the size of the breeding site [48]. In line with reports from other studies [50], *Ae. aegypti* larvae from Nouakchott were found more frequently in habitats with lower water salinity than in those with higher salinity.

Our study has several limitations: firstly, some water collections dried up, resulting in fewer monitoring sessions, and in certain households, access was denied by local residents after an initial observation, limiting further follow up, and secondly, the duration of our study was only one year and could not take into account climatic phenomena such as El Nino or El Nina. Furthermore, although we included a range of environmental variables, additional factors such as organic nutrient content or the presence of predators were not directly assessed. Nevertheless, the *Ae. aegypti* breeding sites were mainly artificial (90%) and therefore independent of these climatic phenomena.

These results underscore the need for targeted management of larval sources in Nouakchott. Public education should emphasize covering water storage and removing unused containers. Due to insecticide resistance, the use of larvicides such as Bti or temephos in key breeding sites is a sustainable method of reducing *Ae. aegypti* populations and preventing dengue epidemics.

## 5. Conclusions

The present study is the first to describe the characteristics of *Ae. aegypti* breeding sites in the urban area of Nouakchott, Mauritania. They correspond mainly to deep, artificial water collections, exposed to shade, and close to dwellings such as water tanks, wells and barrels. In addition, we found that larval abundance was negatively associated with containers of increasing pH and opening surface. As *Ae. aegypti* mosquitoes are established throughout the city of Nouakchott and dengue has become endemo-epidemic since 2014, there is an urgent need to control *Ae. aegypti* populations. Our results could be used by health authorities in charge of vector control to target breeding sites for destruction using different approaches including the use of biological larvicides or the physical elimination of peridomestic breeding sites.

## Figures and Tables

**Table 1:** Main physicochemical characteristics of *Aedes aegypti* breeding sites.

Breeding Site	Number of Observations	Type of Water Collection	pH ^1^	Salinity ^1^ (g/L)	Turbidity ^1^ (ppm)	Temperature ^1^ (°C)	Conductivity ^1^ (µs/cm)
G1	12	Well water storage	8.6, 0.2	0.19, 0.03	219, 33	27.7, 0.8	425, 75
G2	8	Stagnant rainwaterand groundwater	8.3, 0.1	0.90, 0.33	920, 361	26.9, 0.9	1700, 629
G7	14	agricultural wastewater puddle	8.3, 0.1	0.98, 0.07	902, 61	27.8, 1.2	1810, 122
G10	7	agricultural wastewater puddle	8.2, 0.2	0.68, 0.17	642, 157	33.0, 0.6	1283, 313
G15	7	Pipe leak	8.3, 0.2	0.23, 0.07	222, 70	31.1, 1.6	443, 139
G19	6	Fountain bollard drain	7.9, 0.2	0.66, 0.09	632, 73	32.2, 1.2	1266, 147
G23	13	Plastic tank	7.6, 0.2	0.09, 0.01	87, 9	28.2, 0.8	175, 18
G24	13	Plastic tank	7.5, 0.2	0.09, 0.01	96, 7	28.0, 0.7	193, 14
G25	1	Plastic tank	8.0, ND	0.11, ND	119, ND	33.2, ND	238, ND
G29	2	Plastic tank	8.5, 0.2	0.14, 0.06	135, 56	27.4, 1.2	270, 112
G30	5	Plastic tank	8.1, 0.2	0.10, 0.05	119, 46	27.7, 1.3	247, 95
G32	8	Plastic tank	7.7, 0.3	1.17, 1.09	80, 12	27.9, 1.4	159, 24
G33	2	Barrel	8.0, 1.3	0.05, 0.01	52, 11	32.0, 1.5	102, 20
G34	2	Plastic tank	8.0, 0.7	0.05, 0.02	106, 44	31.7, 0.5	213, 91
G35	7	Pipe leak	8.5, 0.1	0.05, 0.01	61, 10	30.0, 1.2	122, 20
G36	2	Well water storage	7.8, 0.3	0.07, 0.01	64, 2	31.4, 2.0	128, 5
G40	4	Well water storage	8.4, 0.2	0.08, 0.03	77, 26	30.8, 1.1	154, 52
G41	4	Plastic tank	8.4, 0.4	0.06, 0.00	61, 1.6	30.2, 1.0	121, 3
G42	3	Plastic tank	7.8, 0.4	0.14, 0.08	131, 73	30.6, 1.0	262, 145
G43	10	Barrel	8.2, 0.2	0.09, 0.02	86, 20	28.2, 1.1	171, 39
G44	6	Barrel	8.0, 0.3	0.10, 0.02	94, 15	27.4, 1.5	188, 30
G45	1	Ablution place	7.1, ND	0.38, ND	362, ND	30.6, ND	723, ND
G46	5	Well water storage	8.3, 0.1	0.12, 0.05	115, 47	30.3, 1.8	230, 93
G47	5	Barrel	7.9, 0.2	0.10, 0.02	102, 17	31.6, 0.5	187, 42
G48	3	Well water storage	9.3, 0.1	0.08, 0.02	82, 25	32.2, 0.7	164, 49
G49	1	Well water storage	8.9, ND	0.15, ND	151, ND	32.3, ND	302, ND
G51	1	Plastic tank	8.2, ND	0.03, ND	52, ND	28.2, ND	102, ND
G52	3	Plastic tank	8.5, 0.1	0.03, 0.02	50, 10	29.3, 1.6	100, 20
G53	7	Plastic tank	8.5, 0.2	0.05, 0.00	55, 3	27.0, 1.1	109, 5
G54	6	Plastic tank	8.5, 0.2	0.06, 0.01	63, 7	30.8, 1.0	125, 14
G55	2	Plastic tank	8.2, 0.0	0.60, 0.10	505, 94	24.3, 4.0	1010, 186
G56	3	Plastic tank	8.9, 0.7	0.09, 0.02	84, 22	24.4, 2.1	167, 44
G58	1	Plastic tank	7.5, ND	0.05, ND	49, ND	24.4, ND	99, ND
G59	1	Plastic tank	8.1, ND	0.24, ND	222, ND	21.5, ND	445, ND
G60	2	Pipe leak	7.4, 0.3	0.19, 0.04	176, 33	24.2, 0.6	346, 72

^1^ Mean and standard error. ND: not determined.

**Table 2:** Multivariate logistic regression with random effect analysis of water collection positivity for *Aedes aegypti* larvae.

Variables		N	P	aOR	CI95%	*p*-Value
Depth (m)	≤0.5	94	28	1		
	>0.5	200	88	5.18	1.66–16.18	0.005
Exposure to the sun	Semi shaded/shaded	188	96	1		
	Sunny	106	20	0.04	0.01–0.26	<0.001
Water collection type	Natural	20	1	1		
	Artificial	274	115	252.88	4.05–15,786.84	0.009
Distance to habitat (m)	≤10	217	114	1		
	>10	77	2	0.00	0.00–0.02	<0.001

N = number of observations; P = number of positive observations for *Aedes aegypti* larvae; aOR = adjusted odds ratio; CI95% = confidence interval 95% of aOR.

**Table 3:** Multivariate binomial regression with random effect analysis of water collection positivity for *Aedes aegypti* larvae.

Variables		N	aOR	CI95%	*p*-Value
pH	<8.3	58	1		
	≥8.3	44	0.50	0.33–0.75	0.001
Size (m^2^)	≤5	26	1		
	>5	76	0.48	0.27–0.87	0.016

N = number of observations; P = number of positive observations for *Aedes aegypti* larvae; aOR = adjusted odds ratio; CI95% = confidence interval 95% of aOR.

## Data Availability

The data presented in this study are available on request from the corresponding author.

## Supplementary Materials

The following supporting information can be downloaded at https://www.mdpi.com/article/10.3390/tropicalmed10060147/s1: Figure S1: Photographs of main *Aedes aegypti* breeding sites in Nouakchott; Table S1: Geographical location of *water collections* in the three Wilaya of Nouakchott; Table S2: Univariate logistic regression with random effect analysis of water collection positivity for *Aedes aegypti* larvae; Table S3: Univariate binomial negative regression with random effect analysis of number of *Aedes aegypti* larvae in breeding sites; Table S4: Number of observations per water collection.

## Abbreviations

The following abbreviations are used in this manuscript:

aOR	adjusted odds ratio
CI	confidence interval
DENV	Dengue virus
