# Supplementary material for: Physical and Chemical Characteristics of Aedes aegypti Larval Habitats in Nouakchott, Mauritania
Source: Trop Med Infect Dis. 2025 May 23;10(6):147. doi: 10.3390/tropicalmed10060147 (PMC12197587; doi:10.3390/tropicalmed10060147)
Supplement: Supplementary file 1 [file tropicalmed-10-00147-s001.zip › Table S1.pdf]

Table S1. Geographical location of breeding sites in the three Wilaya of Nouakchott.

Breeding site	Wilaya	Moughataa	Type of water collection	Latitude	Longitude
G1	NN	Teyaret	Well water storage	18.129284	15.933954 W
G2	NN	Teyaret	Stagnant rainwater and groundwater	18.129492	15.934007 W
G7	WN	Sebkha	agricultural wastewater puddle	18.079816	15.988166 W
G10	WN	Sebkha	agricultural wastewater puddle	18.080936	15.985615 W
G15	WN	Tevragh-Zeina	Pipe leak	18.166040	15.996805 W
G19	WN	Sebkha	Fountain bollard drain	18.077850	15993798 W
G23	WN	Ksar	Plastic tank	18.103429	15.952836 W
G24	WN	Ksar	Plastic tank	18.103696	15.953298 W
G25	WN	Ksar	Plastic tank	18.103034	15.957057 W
G29	SN	Arafat	Plastic tank	18.049987	15.965711 W
G30	SN	Arafat	Plastic tank	18.054631	15.958440 W
G32	WN	Ksar	Plastic tank	18.103598	15.953256 W
G33	WN	Tevragh-Zeina	Barrel	18.094584	15.979966 W
G34	WN	Ksar	Plastic tank	18.102782	15.952660 W
G35	SN	Riyadh	Pipe leak	18.014442	15.954082 W
G36	WN	Tevragh-Zeina	Well water storage	18.098957	15.984908 W
G40	NN	Teyaret	Well water storage	18.129306	15.934194 W
G41	NN	Teyaret	Plastic tank	18.125679	15.934847 W
G42	WN	Tevragh-Zeina	Plastic tank	18.099092	15.984999 W
G43	WN	Tevragh-Zeina	Barrel	18.085128	15.991915 W
G44	WN	Tevragh-Zeina	Barrel	18.085152	15.991297 W
G45	WN	Tevragh-Zeina	Ablution place	18.086216	15.991297 W
G46	WN	Tevragh-Zeina	Well water storage	18.098943	15.974962 W
G47	WN	Tevragh-Zeina	Barrel	18.098823	15.984658 W
G48	WN	Tevragh-Zeina	Well water storage	18.099213	15.985071 W
G49	WN	Tevragh-Zeina	Well water storage	18.098957	15.984908 W
G51	SN	Arafat	Plastic tank	18.054720	15.958407 W
G52	SN	Arafat	Plastic tank	18.054975	15.958498 W
G53	WN	Ksar	Plastic tank	18.102782	15.952660 W
G54	WN	Ksar	Plastic tank	18.103972	15.952027 W
G55	WN	Ksar	Plastic tank	18.129284	15.933954 W
G56	WN	Tevragh-Zeina	Plastic tank	18.115599	15.994126 W
G58	WN	Tevragh-Zeina	Plastic tank	18.084517	15.985854 W
G59	SN	Arafat	Plastic tank	18.054526	15.958833 W
G60	WN	Tevragh-Zeina	Pipe leak	18.090245	15.979273 W

NN: North Nouakchott; WN: West Nouakchott; SN: South Nouakchott.
