# Supplementary material for: Physical and Chemical Characteristics of Aedes aegypti Larval Habitats in Nouakchott, Mauritania
Source: Trop Med Infect Dis. 2025 May 23;10(6):147. doi: 10.3390/tropicalmed10060147 (PMC12197587; doi:10.3390/tropicalmed10060147)
Supplement: Supplementary file 1 [file tropicalmed-10-00147-s001.zip › Table S2.pdf]

Table S2. Univariate logistic regression with random effect analysis of water collection positivity for *Aedes aegypti* larvae.

		N	P	cOR	CI95%	p-value
**Presence of *Culex* larvae**						
	No	189	105	1		
	Yes	105	11	0.51	0.15 – 1.75	0.284
**Presence of *Anopheles* larvae**						
	No	244	111	1		
	Yes	50	5	0.33	0.08 – 1.27	0.107
**pH**						
	< 8.3	148	64	1		
	> 8.3	146	52	0.46	0.44 – 2.65	0.865
**Salinity (g/L)**						
	< 0.18	146	97	1		
	> 0.18	148	19	0.25	0.08 – 0.78	0.017
**Turbidity (ppm)**						
	< 152	147	96	1		
	≥ 152	147	20	0.25	0.08 – 0.77	0.015
**Temperature (°C)**						
	≤ 29.82	147	67	1		
	> 29.82	147	49	0.74	0.30 – 1.81	0.504
**Conductivity (µs/cm)**						
	< 303	147	95	1		
	≥ 303	147	21	0.32	0.10 – 0.98	0.045
**Depth (m)**						
	≤ 0.5	94	28	1		
	> 0.5	200	88	7.45	2.21 – 25.15	0.001
**Size (m^2)**						
	≤ 5	73	21	1		
	> 5	221	95	0.73	0.07 – 7.77	0.792
**Distance to Habitat (m)**						
	≤ 10	217	114	1		
	> 10	77	2	0.00	0.00 – 0.05	< 0.001
**Water collection type**						
	Natural	20	1	1		
	Artificial	274	115	141.17	2.33 – 8537.98	0.018
**Water collection state**						
	Permanent	77	3	1		
	Temporary	217	113	189.24	14.49 – 2471.45	< 0.001
**Exposure to the sun**						
	Shaded/semi shaded	188	96	1		
	Sunny	106	20	0.01	0.00 – 0.18	0.002
**Water colour**						
	Clear	193	110	1		
	Dark	101	6	0.02	0.00 – 0.16	< 0.001
**Plants presence**						
	No	206	107	1		
	Yes	88	9	0.04	0.00 – 0.44	0.008

N = Number of observations; P = Number of positive observations to *Aedes aegypti* larvae; cOR = crude Odd ratio; CI95% = Confidence interval 95% of cOR.
