# Supplementary material for: Physical and Chemical Characteristics of Aedes aegypti Larval Habitats in Nouakchott, Mauritania
Source: Trop Med Infect Dis. 2025 May 23;10(6):147. doi: 10.3390/tropicalmed10060147 (PMC12197587; doi:10.3390/tropicalmed10060147)
Supplement: Supplementary file 1 [file tropicalmed-10-00147-s001.zip › Table S3.pdf]

**Table S3.** Univariate binomial negative regression with random effect analysis of number of *Aedes aegypti* larvae in breeding sites.

| | | N | cOR | CI95% | p-value |
|---|---|---|---|---|---|
| **pH** | | | | | |
| | < 8.3 | 58 | 1 | | |
| | > 8.3 | 44 | 0.50 | 0.32 – 0.77 | 0.002 |
| **Salinity (g/L)** | | | | | |
| | < 0.18 | 92 | 1 | | |
| | > 0.18 | 10 | 1.95 | 0.99 – 3.85 | 0.055 |
| **Turbidity (ppm)** | | | | | |
| | < 152 | 92 | 1 | | |
| | ≥ 152 | 10 | 1.98 | 1.00 – 3.90 | 0.049 |
| **Temperature (°C)** | | | | | |
| | ≤ 29.82 | 63 | 1 | | |
| | > 29.82 | 39 | 0.65 | 0.43 – 1.00 | 0.048 |
| **Conductivity (µs/cm)** | | | | | |
| | < 303 | 91 | 1 | | |
| | ≥ 303 | 11 | 1.78 | 0.92 – 3.46 | 0.089 |
| **Depth (m)** | | | | | |
| | ≤ 0.5 | 26 | 1 | | |
| | > 0.5 | 76 | 0.92 | 0.57 – 1.51 | 0.750 |
| **Size ($m^2$)** | | | | | |
| | ≤ 5 | 26 | 1 | | |
| | > 5 | 76 | 0.49 | 0.26 – 0.91 | 0.025 |
| **Exposure to the sun** | | | | | |
| | Shaded/semi shaded | 85 | 1 | | |
| | Sunny | 17 | 0.66 | 0.31 – 1.38 | 0.270 |

N = Number of observations; cOR = crude Odd ratio; CI95% = Confidence interval 95% of cOR.
