# Supplementary figures and images for: Physical and Chemical Characteristics of Aedes aegypti Larval Habitats in Nouakchott, Mauritania
Source: Trop Med Infect Dis. 2025 May 23;10(6):147. doi: 10.3390/tropicalmed10060147 (PMC12197587; doi:10.3390/tropicalmed10060147)

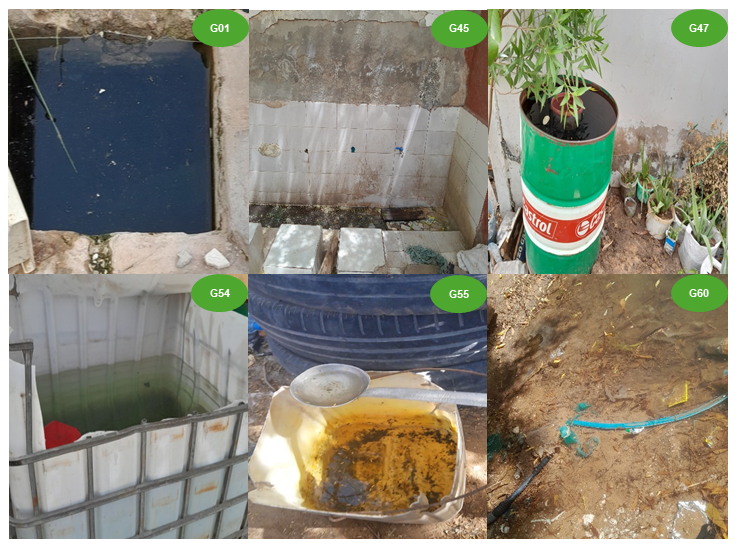

Supplement: Supplementary file 1 [file tropicalmed-10-00147-s001.zip › Figure S1.tif]
